# Supercritical Carbon Dioxide Decellularized Xenograft-3D CAD/CAM Carved Bone Matrix Personalized for Human Bone Defect Repair

**DOI:** 10.3390/genes13050755

**Published:** 2022-04-25

**Authors:** Meng-Yen Chen, Jing-Jing Fang, Jeng-Nan Lee, Srinivasan Periasamy, Ko-Chung Yen, Hung-Chou Wang, Dar-Jen Hsieh

**Affiliations:** 1 Division of Oral and Maxillofacial Surgery, Department of Stomatology, College of Medicine, National Cheng Kung University, Tainan 704302, Taiwan; ccdc0002.tw@gmail.com; 2 Department of Mechanical Engineering, College of Engineering, National Cheng Kung University, Tainan 701401, Taiwan; fangjj@mail.ncku.edu.tw; 3 Department of Mechanical Engineering, Cheng Shiu University, Kaohsiung 833301, Taiwan; jengnan@gcloud.csu.edu.tw; 4 R & D Center, ACRO Biomedical Co., Ltd. 2nd. Floor, No.57, Luke 2nd. Rd., Luzhu District, Kaohsiung 821011, Taiwan; srini@acrobiomedical.com (S.P.); hearty_max@acrobiomedical.com (K.-C.Y.); oscar@acrobiomedical.com (H.-C.W.)

**Keywords:** maxillofacial reconstruction, supercritical CO_2_, collagen bone matrix, 3D carved bone, cytocompatibility

## Abstract

About 30–50% of oral cancer patients require mandibulectomy and autologous fibula reconstruction. Autograft is the gold standard choice because of its histocompatibility; however, it requires additional surgery from the patient and with possible complications such as loss of fibula leading to calf weakening in the future. Allograft and xenograft are alternatives but are susceptible to immune response. Currently, no personalized bone xenografts are available in the market for large fascial bone defects. In addition, a large-sized complex shape bone graft cannot be produced directly from the raw material. We propose the use of porcine bones with 3D CAD/CAM carving to reconstruct a personalized, wide range and complex-shaped bone. We anticipate that patients can restore their native facial appearance after reconstruction surgery. Supercritical CO_2_ (SCCO_2_) technology was employed to remove the cells, fat and non-collagenous materials while maintaining a native collagen scaffold as a biomedical device for bone defects. We successfully developed 3D CAD/CAM carved bone matrices, followed by SCCO_2_ decellularization of those large-sized bones. A lock-and-key puzzle design was employed to fulfil a wide range of large and complex-shaped maxillofacial defects. To conclude, the 3D CAD/CAM carved bone matrices with lock and key puzzle Lego design were completely decellularized by SCCO_2_ extraction technology with intact natural collagen scaffold. In addition, the processed bone matrices were tested to show excellent cytocompatibility and mechanical stiffness. Thus, we can overcome the limitation of large size and complex shapes of xenograft availability. In addition, the 3D CAD/CAM carving process can provide personalized tailor-designed decellularized bone grafts for the native appearance for maxillofacial reconstruction surgery for oral cancer patients and trauma patients.

## 1. Introduction

Bone grafting is an operating technique that has been employed to enhance the healing process by replacing the lost bone with material from the patient’s own body, an artificial, synthetic, or natural substitute, mainly in the fields of orthopaedics, neurosurgery and plastic surgery. Bone grafts are the materials employed for filling cystic defects, for bone fractures and arthrodesis treatment, for traumatic bone defects and for the loss of bone lesions that happens after surgical removal of cancer [1]. Different types of bone grafting materials are currently used, such as autografts, allografts, synthetic variants, xenografts, alloplastic grafts, ceramic-based bone graft substitutes and polymer-based bone graft substitutes [2].

Autologous bone grafting comprises using bone obtained from the same individual recieving the graft. It is considered the “gold standard” for bone grafting because there is less risk of graft rejection, and good osteoinductivity and osteogenicity, as well as osteoconductivity. The drawback of autologous grafts is that they require additional surgery [2,3]. Allografts are obtained from humans and harvested from patients other than the one receiving the bone graft. They need sterilization and deactivation of proteins found in bone, performed using a demineralizing agent such as hydrochloric acid which degrades mineral contents. Xenografts are bone grafts derived from a non-human species, such as porcine and bovine [2]. However, xenografts are neglected because of their highly immunogenic, inadequate biomechanical qualities and foreign body adverse reaction [4]. These drawbacks of the xenografts can be overcome using decellularization techniques, mainly supercritical carbon dioxide (SCCO_2_) which can completely nullify the cons of the xenografts [5].

The SCCO_2_ technology can resolve the drawbacks of the decellularization of bone. Meanwhile, the SCCO_2_ extraction technology works based on using supercritical fluid CO_2_ as the extracting fluid to eliminate the fats, cells and non-collagenous proteins from the bone. The pros of SCCO_2_ extraction technology are that it is natural, safe, non-toxic, non-corrosive, non-flammable, easily accessible and cost-effective. The vital parameters of SCCO_2_ extraction technology are its mild critical coordinates, including pressure at 7.38 MPa and temperature at 31 °C, which can be attained effortlessly and are appropriate for bone materials. The SCCO_2_ technology has been employed for removing lipids from bone, due to its outstanding solvent capacity for lipids; in addition, this technique significantly decreases the antigenicity during bone decellularization while preserving the strength of the bone, similar to that of the original native bone [5]. We have successfully decellularized bone, skin, cartilage and cornea using this SCCO_2_ extraction technology [6]. The biomaterials decellularized using SCCO_2_ extraction technology are highly biocompatible and non-toxic, with enhanced regenerative capability; most importantly without antigenicity and immunogenicity [6,7].

The bone grafts currently available in the market are produced by a technique called high-temperature sintering (300–1300 °C), to completely remove zoonotic infectious and immunogenic agents present in the bone. The high-temperature sintering destructs the intrinsic collagen components in the bone and alters the native porous structures of the bone. In addition, this technique converts the native bone materials into inorganic hydroxyapatite and tri-calcium phosphate, which is much less bioabsorbable relative to native bone [8,9,10].

Oral carcinoma was reported with an incidence rate of 4% of all cancers in the western world [11]. Among the oral cancers, squamous cell carcinoma is the most dominant type of cancer that affects the buccal cavity, and 49% mandibular involvement in cancer patients [12,13]. Oral cancer alters the face by frequently mutilating, and simultaneous cancer resection can lead to overwhelming cosmetic and functional loss leading to psychological, physical, functional and nutritional effects. To overcome this, craniofacial reconstruction is performed, which creates exceptional challenges because of the three-dimensional (3D) shape of the planned construction of the bone for the pivotal significance of re-establishing speech, swallowing, mastication and symmetrical facial contour. Moreover, the results of the reconstruction are frequently unpredictable and learning-curve dependent, making pre-operative scheduling tough for reconstructive surgeons [13,14].

The conventional protocol for maxillofacial bone grafting includes multifaceted stages which are expensive, time-consuming, extremely traumatic for the patient and depend on the skills of the maxillofacial surgical team [15]. The intricacy of conservative maxillofacial prosthodontics manufacture needs many weeks and many clinic visits by the patient to try the grafts, and functional and aesthetic adjustments [16]. Most patients do not opt for subsequent surgical correction, and the degree of their defects induces a lack of self-confidence, impairing their daily activities and social lives [17]. Computer-aided design (CAD) is an innovative technology that offers advantages in the development and execution of inspiring bone graft production for traumatic and oncological craniofacial reconstructions [18]. CAD is a multifaceted and sophisticated pre-operative course. CAD starts with the acquisition of a patient’s craniofacial skeleton with a 3D rendered, 64-slice high-resolution CT scan [17,19,20,21]. In the production of 3D customized scaffolds using numerous materials and techniques, with precise imaging methods, the design and construction of CAD-CAM scaffolds has become a straightforward method [19]. Investigations involving the customised scaffolds for alveolar ridge augmentation revealed well established positive outcomes [20,22,23].

The purpose of the current study was to evaluate the SCCO_2_ decellularized -CAD-CAM facilitated 3D Lego jaw bone production and to assess its in vitro biocompatibility. Most of the previous studies focus on the production of a certain part of the jaw. However, in the present study, we hypothesise producing the whole jaw using SCCO_2_ decellularized porcine bone matrices to construct a Lego form of the complete jaw.

## 2. Materials and Methods

### 2.1. 3D Mandibular Model Construction

**Figure 1:**
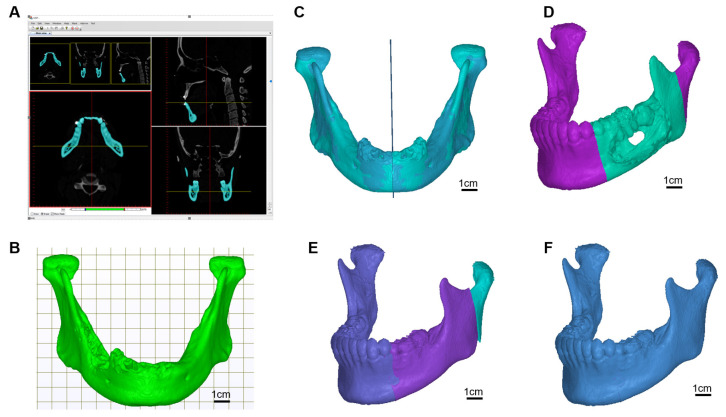
Scan image deciphering and 3D image generation. (**A**) Imaging decoding and model reconstruction software, (**B**) mandible model, (**C**) find the optimal symmetry plane and mirrored model, (**D**) locate the cancer region, (**E**) replace the cancer region with region mirrored part, (**F**) target template.

The patient was diagnosed with a tumour located in the oral cavity. Computerized tomography (CT) from GE MEDICAL SYSTEMS with 1 mm slice thickness was used. As shown in Figure 1A,B, the DICOM imaging was decoded first and then reconstruct to a three-dimensional mandibular model by our-developed software. The OSP (Optimal Symmetry Plane) was automatically generated by locating the one and only symmetry plane which contains the maximal voxel pairs of the collateral parts [24]. The OSP was used for mirroring the right and left sides of the mandible to the other side (Figure 1C). Surgeons determined the region of the tumour located on the mandible (Figure 1D), and the mirror mandible was used to replace the tumour region (Figure 1E). Since mild facial asymmetry is common in adults, a gap usually exists between the condyle processes from the original left and the mirrored right. To adjust the positions of the mirrored half-right mandible model on-screen to overlap the original condyle processes on the left side, a complete symmetry mandible template was created (Figure 1F). If the tumour was located across the centre part of the mandible, a database of the mandibular model from patients who took orthognathic surgery was involved. The smoothing technique was then applied to generate C^2^ continuity between the donor and the replacement parts.

**Figure 2:**
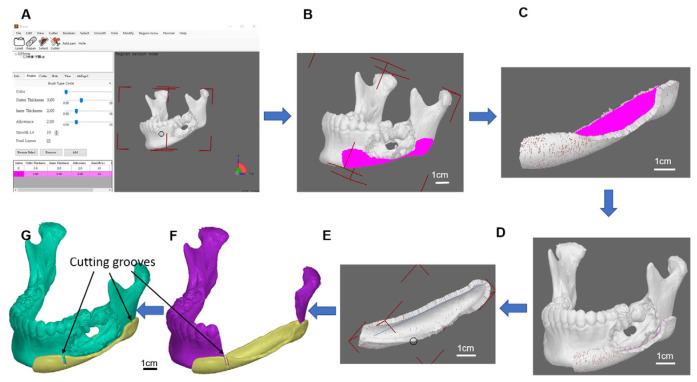
Osteotomy guide model. (**A**) Our developed osteotomy guide software, (**B**) container region, (**C**) generates a container with a selective thickness, (**D**) defect mandible with the container, (**E**) container refinement, (**F**) grooves of the resection guide, (**G**) cutting guide with the defect mandible model.

*The resection guide and puzzle container:* The osteotomy guide is mainly used to assist the tumour resection. The original mandible model was invoked for further resection guide, puzzle parts, and its container design. A guide plate with a selective thickness was generated on the surface of region indication by our-developed software, to cover the region of resection on the mandible decided by the surgeon (Figure 2). Two ends of grooves for bone sawing were made on the container to indicate the edges of resection. It was provided for the surgeon to be used during operation in assisting precise tumour resection. Functions of the resection guide were not only used for precise resection but also to ensure the implanted bone grafts puzzles were securely placed and reform the curved shape of the mandible in origin.

### 2.2. Osteotomy Guide Fabrication and Multi-Piece Puzzle Setup

**Figure 3:**
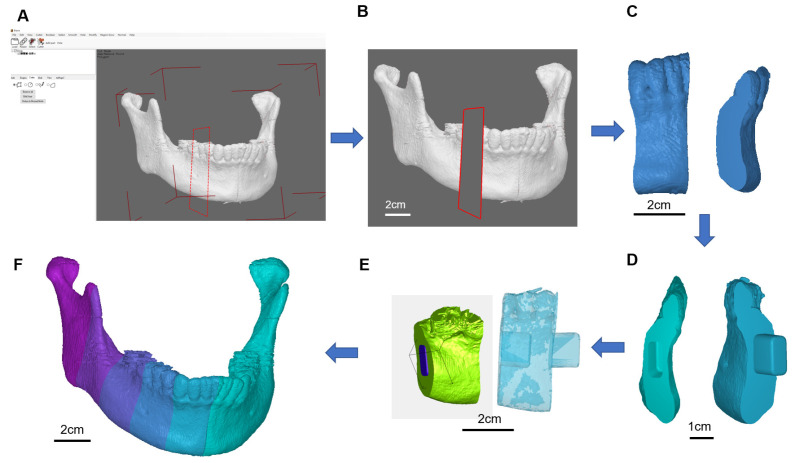
Multipiece Lego design. (**A**) Multi-piece design software, (**B**) selective length of one of the puzzle pieces, (**C**) puzzle piece, (**D**) latched puzzle pieces, (**E**) model views with and without transparency, (**F**) full mandible with 6 puzzle pieces on the right side of the mandible model.

**Figure 4:**
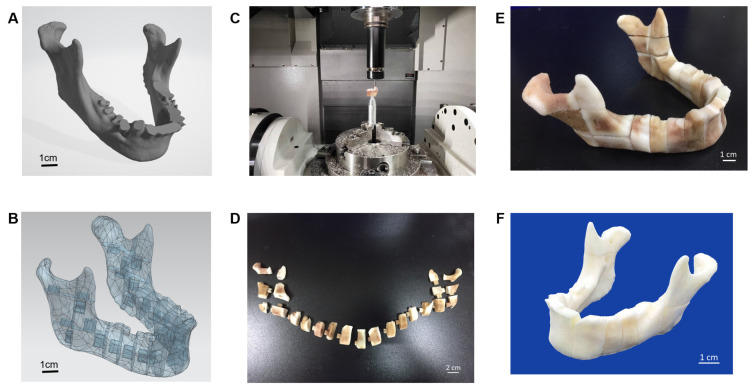
Five-axis CNC machining for the 3D bone for the defect repair, (**A**) STL file of the jaw bone, (**B**) geometric model of jaw bone Lego design by multiple splicing, (**C**) Five-axis CNC machining of the bone, (**D**) Lego jaw bone before arranging, (**E**) Lego jaw bone after arranging, (**F**) Lego jaw bone after cleansing with hydrogen peroxide.

To provide precise tumour resection and bone grafts placement, the developed software was able to construct connection latches to fasten bone graft puzzles together. The target template for the defect replacement region was segmented into many pieces of lengths of less than 2 cm which allow bone fusion between the cutting face of the mandible and the porcine bone grafts (Figure 3). Many pieces of bone grafts work like a curved combination of many pieces of puzzles to form a template mandible, latching in between connect one groove and one hole at the two-end faces attached to each piece (Figure 4). The prerequisite is that such customized materials and fabricated bone pieces must meet the requirements of the quality measurement system of classes II and III. The tumour resection guide was categorized as class II, which was fabricated by the 3D printer and bio-compatible resin.

### 2.3. Cutting and Milling a Set of Multi-Piece Lego Assembly for Defect Replacement

The porcine bone block was the basic material of the carved bone matrices. A5-axis Computer Numerical Control (CNC) CT-350 machine integrated with the controller, SIEMENS 840Dsl, was used to manufacture the carved bone puzzles. The Productivity + ™ Active Editor Pro tool probe software was employed for measurement path planning in pre-processing. The process was to check both the remaining quantity after machining and machining accuracy and efficiency. At first, a special design of bone matrices metal jig was fabricated by the CNC machine. Since the porcine material is porous and brittle, the complex geometric shape increases the complicatedness of machining. Therefore, a series of associated numerical control parameters to be used in the CNC milling should be carefully examined. We developed an associated five-axis post-processing programming control to handle such complicate applications. A template mandible with 23 connective pieces was selected to fulfil the prototype. To evaluate the accuracy of the design, we used the Renishaw OMP400 line measurement system for accuracy before assembling it into a complete mandible.

### 2.4. Porcine Bone Preparation

Porcine bone was purchased from Tissue Source LLC. The bones were subjected to preliminary processing such as the removal of peripheral muscle tissue and fat. The porcine bone was washed with phosphate-buffered saline (PBS). The bone was 3D CAD/CAM cut to an appropriate size according to the process design, and the skull bone block was subjected to the SCCO_2_ process and further testing.

### 2.5. The SCCO_2_ Decellularization of Bone Blocks

The bone blocks were placed on a tissue holder and placed inside a SCCO_2_ vessel system (Helix SFE Version R3U, Applied Separations Inc., Allentown, PA, USA) with a co-solvent of 60–95% ethanol. The SCCO_2_ process was operated at 350 bar and 35–40 °C for 80 min to decellularize the bone blocks.

### 2.6. Fat Analysis of SCCO_2_ Decellularized Porcine Bone Blocks

Bone sample 100 mg powder was added to 500 µL filtered Oil red O (2.1 mg/mL) solution and incubated at room temperature for 15 min. The Oil red O solution was washed off by vortexing with distilled water twice. An amount of 250 µL of isopropanol (100%) was added to the washed sample, vortexed and incubated at room temperature for 5 min. The Blank was performed without a bone sample. The supernatant, 100 µL was taken to measure the fat in a microplate reader at 490 nm.

### 2.7. Hematoxylin and Eosin Staining of SCCO_2_ Decellularized Porcine Bone Blocks

Haematoxylin stains the nucleus of the cell displays a deep blue-purple colour, and eosin stains the protein of cytoplasm and extracellular matrix a pink colour. The native bone and decellularized bone blocks were fixed in 4% buffered formaldehyde at room temperature overnight, dehydrated stepwise and embedded in paraffin. The paraffin-embedded bone sections were cut to a thickness of 5 μm, mounted on the glass slides, fixed and stained with haematoxylin and eosin (H&E) to assess the completion of decellularization. Photographs were recorded under a microscope (Olympus bx53) for further evaluation.

### 2.8. Collagen Staining by Masson’s Trichrome Stain of SCCO_2_ Decellularized Porcine Bone Blocks

After fixing and dehydrating the decellularized bone blocks with 4% formaldehyde, the paraffin-embedded bone sections were cut to a thickness of 5 μm, mounted on the glass slides, fixed and stained with Masson’s trichrome stain to observe the collagen distribution in the bone.

### 2.9. Collagen Quantification by Estimating Hydroxyproline of SCCO_2_ Decellularized Porcine Bone Blocks

The concentration of collagen in the bones was analysed by estimating the hydroxyproline content using (the BIO VISION) reagent to quantify the collagen content of the bone sample. The bone samples were homogenized by adding 100 µL of ddH_2_O and then 100 µL of HCl (~12N) for hydrolysis, incubated at 120 °C for 3 h and centrifuged at 1000× *g* for 3 min. Chloramine T was allowed to stand at room temperature for 5 min and 100 µL of DMAB was added for 90 min at 60 °C. The hydroxyproline standard was diluted to 0.1 mg/mL, and 0, 2, 4, 6, 8 and 10 µL were taken out and the standard curves of hydroxyproline concentration of 0, 0.2, 0.4, 0.6, 0.8 and 1 mg/mL were drawn; then the samples measured by 560 nm in a microplate reader.

### 2.10. DNA Quantification and Agarose Gel Electrophoresis of SCCO_2_ Decellularized Porcine Bone Blocks

The bone blocks (n = 3 each) were processed and extracted for genomic DNA employing a commercially available kit (NautiaZ Tissue DNA Mini Kit, Nautiagene). The DNA extracted was measured employing a microplate reader at 260 nm. The amount of residual DNA and fragment size in the bone blocks were determined by agarose gel electrophoresis.

### 2.11. Electron Microscopy of SCCO_2_ Decellularized Porcine Bone Blocks

The fixed and dehydrated bone block samples were air-dried in a clean chemical hood. The dried bone samples were glued to the aluminium table using a carbon double tape, and then the gold particles were sprayed using Hitachi E1010 Ion Sputter for scanning electron microscopy using Hitachi S-3400N scanning electron microscope for observation. Photomicrographs were recorded for analysis.

### 2.12. Stress Analysis of the Decellularized Bone Blocks

The biomechanical testing was performed using an MTS testing machine (MTS 858 Mini Bionix^®^ II Biomaterials Testing System, Eden Prairie, MN, USA). The decellularized bone block samples were placed on a specially designed platform with a self-aligning function to ensure vertical compression. The sampling frequency was set at 500 Hz with a probe diameter of 2.0 mm. A pre-load of 2 Mpa with 30 s of accommodation time followed by a continuous and progressive load at a rate of 2 mm/min was applied. The first peak force detected during the test was recorded as the ultimate strength. The displacement versus force was recorded to calculate the maximal load. Maximal load in Newtons, stiffness in Newtons/mm and energy to the maximal load in millijoules were measured and recorded.

### 2.13. Cell Adhesion and Growth Analysis on the Decellularized Bone Blocks by SEM

Decellularized bone blocks were placed in 24 well-plates. The cell density of MG63 cells was 1 × 10^6^ cells/mL was added 1ml of cell solution per well directly onto the bone blocks. After overnight (16h) incubation, the samples were washed with PBS several times, then transferred to a new 24 well-plate, 2ml DMEM-10% FBS medium was added for culturing. After Day 4, the samples were washed with dPBS several times, then transferred to a new 24 well-plate and 2 mL of DMEM-10% FBS medium was added to continue the culture. On Day 7, the samples were washed with PBS several times, then transferred to a new 24 well-plate, 2.5% glutaraldehyde was added and fixed overnight (16 h) at 4 °C. Samples were then dehydrated in 50%, 60%, 70%, 80%, 90% and 95% ethanol for 15 min each, followed by 99.5% ethanol for 15 min twice and freeze-dried. Bone blocks were fixed on carbon tape and sprayed with platinum for SEM scanning.

## 3. Results

### 3.1. 3D Tissue Model Reconstruction and Lego Jaw Bone Carving Using 5 Axis CNC Machine

The CT scan image was decoded, and the mandibular jaw was reconstructed using model reconstruction software. The mandible was modelled to find the optimal symmetry of the plane and a mirror model was generated. The tumour area in the jaw was located and the cancer region was replaced with the mirrored area. Finally, the template of the mandible was created (Figure 1).

Using our in-house osteotomy guide software, the container region was designed and the container region modelled. In addition, the thickness of the container was selected as the option in the software. The defect mandible with the container was aligned and modelled, and the refinement was carried out. The cutting grooves were generated using the software. Finally, the cutting guide with the defect mandible was modelled (Figure 2).

The multi-piece design software was employed to select the length of individual pieces along with puzzle pieces including latched puzzle pieces. To finish, we can visualize the model views with and without transparency. All the designed individual pieces of a puzzle piece and latched puzzle pieces were designed to form a full mandible with six puzzle pieces on the right side of the mandible model (Figure 3).

We used the Geomagic Studio software to delete and remove the abnormal grid and reconstruct it into an IGES file. After the STL file was transferred to the IGES, it was integrated using the NX software, and the geometry of the defect was divided into multiple heterogeneous bones in a Puzzle-to-Latch design/lock and key design. Finally, the pieces were carved using a five-axis CNC machine into 23 pieces and cleansed using hydrogen peroxide to form a complete jaw (Figure 4).

### 3.2. Characterization of SCCO_2_ Derived Bone Blocks

#### 3.2.1. Fat Content

The results of the fat content of the SCCO_2_-processed bone blocks showed an optical density of 0.357 ± 0.024 (n = 3) at 490 nm, which is well below the acceptable range of <0.6, therefore the SCCO_2_-processed bone blocks were qualified to be a medical device according to ISO standards.

#### 3.2.2. H&E Staining

**Figure 5:**
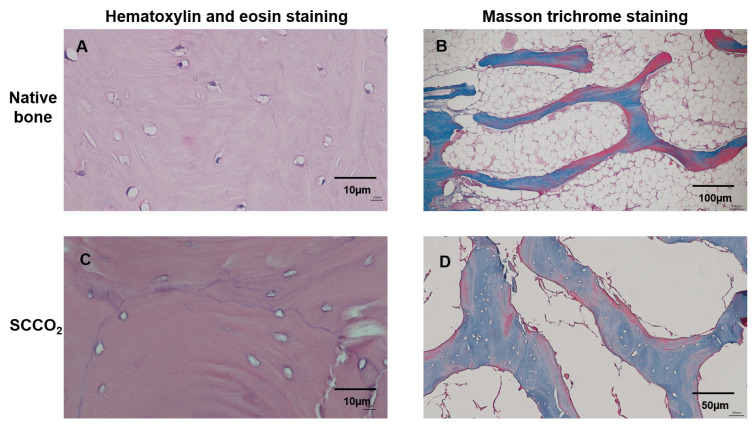
Characterization of SCCO_2_-processed bone blocks by haematoxylin and eosin staining and Masson trichrome staining of the native bone block (**A**,**B**) and SCCO_2_-processed bone blocks (**C**,**D**).

The H&E staining of the SCCO_2_-processed bone blocks showed no nucleus or cell debris indicating complete decellularization, whereas nucleus and cell staining was visible in the native porcine bone Figure 5A. The results show that the decellularization process can effectively remove the cells in the bone tissue Figure 5C.

#### 3.2.3. Masson Trichrome Staining

The decellularization of the bone blocks and retaining ECM of the bone tissue were evaluated by Masson trichrome staining. The native porcine bone contained cells and collagen were stained (Figure 5B), whereas the SCCO_2_-processed bone blocks showed no cells; however, all the collagen was well preserved (Figure 5D), indicating the SCCO_2_ process did not alter the ECM and collagen of the bone tissue. The collagen content was quantified in the SCCO_2_-processed bone blocks showed 293.37 ± 22.49–335.07 ± 82.11 µg/mg of bone decellularized bone tissue with a percentage of 29–34%.

#### 3.2.4. Residual DNA Content

**Figure 6:**
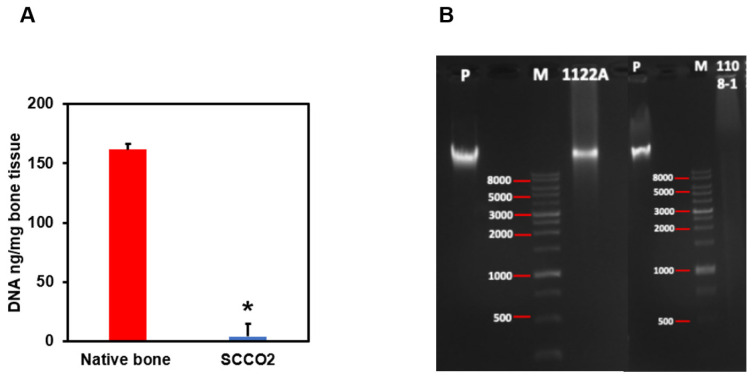
Characterization of SCCO_2_-processed bone blocks by DNA quantification (**A**) and agarose gel electrophoresis (**B**). Results were expressed as mean ± SD, * *p* < 0.001 were considered statistically significant for different tests (N = 3).

The content of DNA was analysed by quantification and agarose gel electrophoresis to confirm the decellularization of the bone blocks by SCCO_2_ technology. Three minimum criteria for optimal and successful decellularization are: (1) tissue samples should contain < 50 ng of dsDNA per mg of dry ECM, (2) any remaining DNA fragments should be smaller than 200 base pairs, and (3) the tissue should not have visible nuclear material when stained with DAPI or haematoxylin and eosin. DNA quantification of SCCO_2_-processed bone blocks showed an average of 4.17 ng/mg of DNA (Figure 6A) which is well below the permissible level of 50 ng/mg residual DNA content for medical implant devices according to the Biological Evaluation of Medical Devices—Part 1 (ISO 2018). In addition, agarose gel electrophoresis revealed no DNA band in the SCCO_2_-processed bone blocks, whereas DNA bands with fragment length were detected in the native porcine bone Figure 6B.

#### 3.2.5. Characterization of SCCO_2_-Processed Bone Blocks by SEM

**Figure 7:**
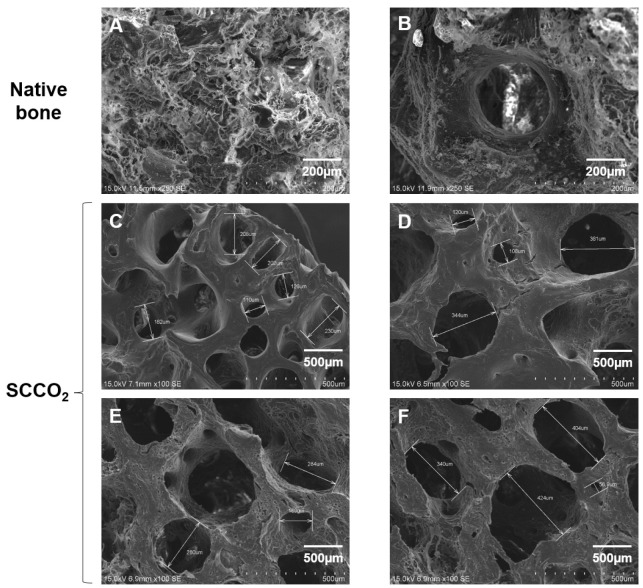
Characterization of SCCO_2_-processed bone blocks by scanning electron microscopy of the native bone block (**A**,**B**) and SCCO_2_-processed bone blocks (**C**–**F**).

To study the surface morphology of SCCO_2_-processed bone blocks, we analysed the SEM images. The native porcine bone shows cells and cellular debris on the surface of the bone (Figure 7A,B). The SCCO_2_-processed bone blocks showed no residual cells and soft tissue, and retained the natural pore structure ranging from 100~400 µm (Figure 7C–F) in the bone tissue which is essential for angiogenesis and enhances bone growth. Furthermore, SCCO_2_-processed bone blocks contained micropores of diameter < 10 μm, which are essential for the transport of body fluids, ion transportation and the attachment of osteoblast suitable for bone regeneration.

#### 3.2.6. Mechanical Strength of SCCO_2_-Processed Bone Blocks

To study the strength and stiffness of SCCO_2_-processed bone blocks, we analysed the compression test. The test results of SCCO_2_-processed bone blocks of different sizes showed that they were all greater than the acceptable standard of 2 MPa (Table 1).

#### 3.2.7. Cell Adhesion and Growth on SCCO_2_-Processed Bone Blocks by SEM

**Figure 8:**
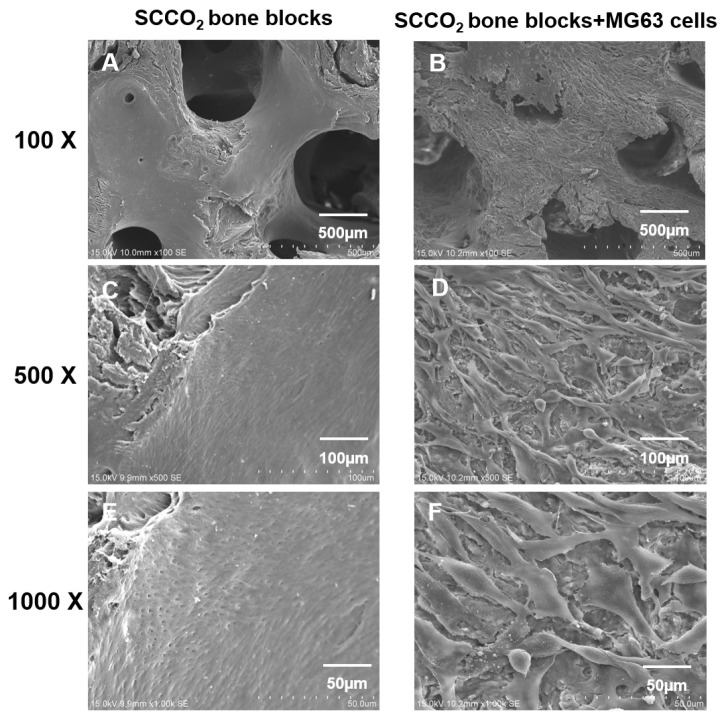
Characterization of SCCO_2_-processed bone blocks by in vitro cell adhesion and growth on the surface via scanning electron microscopy of the native bone block (**A**,**C**,**E**) and SCCO_2_-processed bone blocks (**B**,**D**,**F**).

**Figure 9:**
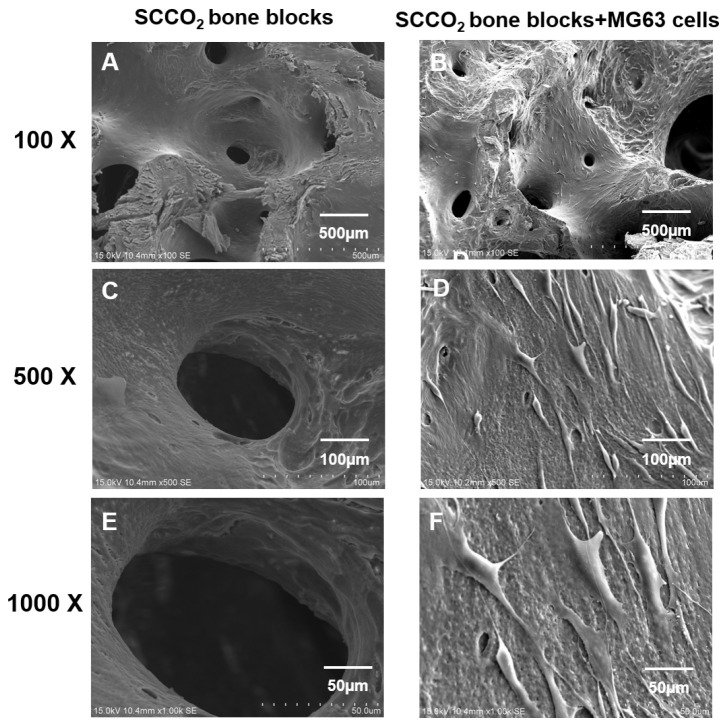
Characterization of SCCO_2_-processed bone blocks by in vitro cell growth inside the pores via scanning electron microscopy of the native bone block (**A**,**C**,**E**) and SCCO_2_-processed bone blocks (**B**,**D**,**F**).

SEM was performed to evaluate the cell adhesion surface, cell viability and growth of MG63 cells on SCCO_2_-processed bone blocks. The results show good cell adhesion of MG63 cells on SCCO_2_-processed bone blocks. In addition, MG63 cells grow well on the surface of the SCCO_2_-processed bone blocks, the cells were also found to grow inside the pores of SCCO_2_-processed bone blocks. Overall, the SCCO_2_-processed bone blocks showed good cell affinity, adhesion and growth even inside the pores after seeding of MG63 cells, which proves excellent cytocompatibility (Figure 8 and Figure 9).

## 4. Discussion

Porcine bone xenografts are demonstrated to be an outstanding grafting material for bone augmentation surgeries. The porcine cortical bone used in clinical studies revealed exceptional results and promising clinical application [25]. Already we established the regenerative capability of porcine grafting material such as the cornea, skin matrix and bone can be enhanced by decellularization by employing SCCO_2_ extraction technology [26,27,28] The novel SCCO_2_ extraction technique was used to proficiently eliminate the cellular elements of porcine grafting material such as cornea, skin matrix and bone and the biomechanical and biomechanical properties of the graft are well conserved. Furthermore, the decellularized graft material exhibited no immunological reactions, with good biocompatibility and long-term stability [6,7,29,30]. Therefore, in the present study, we used SCCO_2_ extraction technology to decellularize the porcine bone after 3D CAD/CAM carving and created a Lego set of the lower jaw, which possesses good biocompatibility and can be used in oral cancer patients where part or full jaw can be replaced.

The bone deformities of the craniofacial skeleton are complex deformities due to congenital abnormalities or from trauma, infection and tumour resection [31]. The reconstruction of these craniofacial bone abnormalities is problematic as these abnormalities are three-dimensional (3D) and complex in size and shape. Until recently, the bone defects have been reconstructed with bone grafts and alloplastic implants. The gold standard for facial reconstruction is the use of autogenous bone [32]. The carving of the bone grafts is performed freehand, which is linked to issues of poor fit within the bone-implant interface, irregular contours, palpable edges and persistent asymmetry [33]. Therefore, in the present study, to obtain a precise bone graft fit with bone interface contact the 3D of the CT image was performed, subsequently mirroring the facial skeleton and finally, modelling and swift prototyping of the left mandibular jaw region were performed.

In the present study, SCCO_2_-processed bone blocks were completely decellularized, with the removal of fat and DNA. However, the native collagen scaffold of the SCCO_2_-processed bone blocks remains intact and unaltered. Several bone grafts that are accessible in the market are manufactured by a high-temperature sintering process (300–1300 °C), which destroys the collagen scaffold of the bone graft. The conventional decellularization methods employing chemical etching and oxidation methods eliminated only 61% of DNA in porcine decellularized patellar tendons samples [34], and 90% in porcine cartilage and bone [35]. However, the SCCO_2_ decellularization method successfully washed off 99.1% DNA in the bone blocks, which has an excellent advantage over the traditional decellularization methods.

In the present study, the SEM revealed that the SCCO_2_ process is mild and does not change the native porous structure of bone. The porous nature of the bone plays a vital role in cell seeding diffusion and the mechanical strength of bone graft [36]. The extreme porous nature of the bone graft helps vascularization, osseointegration from native adjacent bone, osteoblast and osteoclast infiltration to facilitate bone remodelling [37]. The porous nature of the bone graft aids the development of vascular networks to induce the proliferation and differentiation of osteoblasts and enhance the ingrowth of new bone within the bone graft [38]. The SCCO_2_ process also preserves the diverse range and network of pores from micro- to nanosize which are indispensable in angiogenesis and encourage both bone growth and reorganization in and around the bone graft [39]. The SCCO_2_ porcine bone graft X-ray diffraction studies revealed an excellent Ca/P ratio of 1.75 as measured employing the wavelength dispersive X-ray fluorescence study, demonstrating the SCCO_2_ technology preserved the original chemical composition of bone graft which correlates with high levels of Ca and P induces osteogenesis [40].

In the present investigation, SCCO_2_-processed bone blocks revealed excellent cell adhesion and cytocompatibility with the MG63 cell line examined through SEM. Medical device contamination is routinely assessed which includes the biocompatibility study to appraise the pyrogenic potential of a fever-inducing nature of the product [41]. Our previous SCCO_2_-processed bone graft studies revealed no signs of adverse effects in cytotoxicity studies in mouse fibroblast L929 cells [6]. The SCCO_2_-processed bone graft is evidenced to be pyrogen-free. In addition, the SCCO_2_-derived bone graft is mutagen free, which is evidenced by in vitro gene mutation analysis in L5178Ytk+/− cells. In vivo, systemic toxicity analyses are routinely performed to evaluate a medical device’s toxicity related to organs including the liver, heart, kidneys and brain [42]. In conclusion, the SCCO_2_-processed bone graft proved to be non-toxic and do not have any sign of adverse effects, no mortality and no obvious gross lesions were detected [6,7].

In the current study, SCCO_2_-processed bone blocks depicted good cytocompatibility along with excellent stiffness in the biomechanical analysis. The conventional decellularization process by chemical detergent and biological agents altered the ECM, the SCCO_2_-processed bone graft preserved ECM and its mechanical properties [43]. Our previous SCCO_2_-processed bone graft studies revealed good biocompatibility, healing and bone regeneration in the osteochondral defect model in rabbits. The SCCO_2_-processed bone graft filled in the defects performed in the distal femoral metaphysis of rabbits, which acts as a bone substitute with absorbable calcium salt and proved to regenerate the bone void with new bone formation indicating SCCO_2_-processed bone graft possesses excellent osteoconductivity and regenerative potential [6]. The dog’s mandibular extraction socket of SCCO_2_-processed bone graft depicted good bone regeneration was evaluated in our lab. Newly formed bone showed greater stiffness relative to the Bio-Oss^®^-treated sites in the biomechanical analysis. The SCCO_2_-processed bone graft depicted more new bone formation and improved bone bridging, than the Bio-Oss^®^ treatment [7].

The clinical studies involving ABCcolla^®^ Collagen Bone Graft which is a SCCO_2_-processed bone graft from our research group is a strong implant covering the orbital defect with low complication rate, low infection rate, low cost, high biocompatibility and high osteoconductive function [30]. However, preclinical studies depicted that SCCO_2_-processed bone graft exhibits excellent bone remodelling, regeneration and reabsorption [6,7]. The first clinical study involving orbital wall reconstruction with ABCcolla^®^ Collagen Bone Graft implantation revealed the potential application of the xenograft de-cellular framework to regain functions of orbital walls [30].

In the present study, we used CAD/CAM workflow to reproduce the lower jaw from 3D carving SCCO_2_-processed Lego bone blocks. End-stage cancer involving the jaws often needs segmental resection. Subsequent reconstruction by free microvascular bone transfer is based on the choice in patients with acceptable health status [44,45]. The CAD-based planning and preoperative manufacturing by CAM of surgical templates to support the surgery of tumours. The CAD-CAM workflow permits the preoperative description of cutting paths and angles at the resection site, modelling of the bone graft as well as the shape of the bone graft leading to an efficient composable and placeable reconstruction. The complete assembly time including the intraoperative cutting, positioning and refinement of the bone graft is decreased by the CAD/CAM workflow [46,47], thereby significantly decreasing the issue associated with long surgery time. In addition, CAD-CAM workflow improves the aesthetic and functional outcome by augmenting the position and contour of the reconstruction [44,46]. In the present study, we CAD-CAM carved the porcine bones into pieces of Lego to form the whole jaw and processed by SCCO_2_ decellularization. To our knowledge, this is the first attempt to create a complete jaw, and it is ready to move into the initial stage of clinical application.

## 5. Conclusions

This section is not mandatory but can be added to the manuscript if the discussion is unusually long or complex.

## Figures and Tables

**Table 1:** Characterization of SCCO_2_-processed bone blocks by mechanical stiffness.

SCCO_2_ Derived Bone Blocks	Mechanical Stiffness (MPa) Mean ± SD
2 × 3 × 2 cm^3^	13.75 ± 3.99
2 × 2 × 2 cm^3^	11.34 ± 1.74
2 × 1 × 2 cm^3^	3.46 ± 3.04
1 × 1 × 2 cm^3^	12.18 ± 1.40

## Data Availability

Not applicable.
